# CUMS Promotes the Development of Premature Ovarian Insufficiency Mediated by Nerve Growth Factor and Its Receptor in Rats

**DOI:** 10.1155/2020/1946853

**Published:** 2020-06-30

**Authors:** Xiaoyan Fu, Qun Zheng, Ning Zhang, Mingxing Ding, Xiaoming Pan, Wenqian Wang, Haohao Chen

**Affiliations:** ^1^Medical Molecular Biology Laboratory, Medical College, Jinhua Polytechnic, Jinhua, Zhejiang Province, China; ^2^Center of Clinical Reproductive Medicine, Jinhua People's Hospital, Jinhua, Zhejiang Province, China

## Abstract

This study aimed to investigate whether chronic unpredictable mild stress (CUMS) affects follicular development in ovaries through the nerve growth factor (NGF)/high affinity nerve growth factor receptor, the Tropomyosin-related kinase A (TrkA) receptor, mediated signaling pathway and to reveal the relationship between chronic stress and premature ovarian insufficiency (POI) development. In this experiment, a CUMS rat model was constructed. It was found that serum estradiol (E2), anti-Mullerian hormone (AMH), and gonadotropin-releasing hormone (GnRH) levels decreased, while follicle-stimulating hormone (FSH) levels increased. The expression of NGF, TrkA, p75, and FSHR in ovarian tissue decreased significantly. The expression levels of TrkA and p75 protein in ovarian stroma and small follicles were observed by an immunofluorescence assay. In addition, the numbers of small follicles were significantly reduced. The expression of TrkA, p75, and FSHR in CUMS ovarian tissue was upregulated by exogenous NGF *in vitro*. Furthermore, after treatment with NGF combined with FSH, E2 secretion in ovarian tissue culture supernatant of CUMS rats also increased significantly. Therefore, CUMS downregulates NGF and TrkA and promotes the occurrence of POI in rats. Exogenous NGF and FSH can upregulate the NGF receptor, E2, and AMH *in vitro*, and improve the rat ovarian function. Future studies may associate these results with female population.

## 1. Introduction

In recent years, premature ovarian insufficiency (POI) has become an increasingly prominent problem in the field of female reproductive health. POI is defined as amenorrhea due to the cessation of ovarian function before the age of 40. Patients with POI often exhibit high levels of follicle-stimulating hormone (FSH) and low estradiol (E2) levels [1, 2]. Early loss of ovarian function has a significant impact on the psychosocial and physiological health of patients with POI.

Previous studies have shown that a variety of pathogenic mechanisms may lead to the development of POI, including chromosome, heredity, autoimmunity, metabolic (galactosemia), infection (mumps), and iatrogenic causes. Multiple pathogenic factors are associated with the pathogenesis of POI, and there are no effective treatment methods available at present [3]. Recent studies have shown that the occurrence of POI is closely related to anxiety, depression, and other negative emotional states [4]. Approximately, 43% of patients with POI have been found to have a history of depression, and about 26% of them have depression within five years before being diagnosed with POI [4]. At present, there are few studies on the effect of psychological stress on ovarian function. However, some animal studies have found that chronic unpredictable mild stress (CUMS) can lead to the decrease of ovarian reserve in C57BL/6 mice [5] and SD rats [6]. These studies show that stress does have an effect on ovarian function. Psychological stress can inhibit and impair the female reproductive and endocrine functions. However, the mechanism of how psychological stress leads to the decrease of ovarian reserve, even to ovarian dysfunction or even premature ovarian failure is not clear. Nerve growth factor (NGF) is a member of the neurotrophic factor family. It plays an important role in maintaining the activity and proliferation of nerve cells and nonnerve cells. NGF and its receptors play an important role in follicular development. It activates its function by binding two kinds of receptors: high-affinity receptor Tropomyosin-related kinase A (TrkA) and low-affinity receptor p75. It has been found that NGF and its receptors are expressed in different types of ovarian cells (oocyte, granulosa, theca, and interstitial cells) in different species, including human, rodent, bovine, ovine, and caprine species [7]. NGF is essential for early follicular development. The mechanism of NGF supporting follicular growth includes not only the proliferation signal of mesenchymal cells but also the induction of FSH receptor [8]. NGF-knockout rat studies have confirmed that NGF is an important factor for primordial follicular growth in the nongonadotropin-dependent phase. NGF effects follicle survival in a dose-dependent manner *in vitro* and can induce follicle-stimulating hormone receptor (FSHR) expression [9].

In our previous studies, chronic unpredictable mild stress significantly reduced the number of primordial follicles and primary follicles in SD rats and decreased ovarian reserve. However, is NGF and its receptor related to the reduction of ovarian reserve caused by chronic stress? Although it has been confirmed that chronic stress affects follicular development through neurotrophins in the ovary, there is no consistent conclusion about the effect of chronic stress on NGF. After 4 weeks of chronic intermittent cold stress, NGF protein in ovary of adult SD female rats increased, but the mRNA concentration of NGF and its low-affinity receptor (p75) did not increase significantly [10]. However, the expression of NGF mRNA decreased in the ovaries of offspring rats suffering from chronic intermittent cold stress during pregnancy, but the expression of p75 receptor mRNA did not change [10].

Chronic cold stress can cause the activation of sympathetic nerves in the ovaries and downregulate the expression of NGF and high-affinity nerve growth factor receptor (TrkA). Therefore, the expression of FSHR is negatively regulated due to the downregulation of NGF expression, which leads to follicular dysplasia [10]. In contrast, another study has found that the expression of NGF protein increases after chronic cold stress, with no changes in the mRNA levels of NGF and p75 [10]. These results indicate that there is a correlation between chronic stress and NGF and its receptors. Chronic stress may affect the growth and development of ovarian follicles through signal transduction pathways mediated by NGF and its receptors.

Chronic unpredictable mild stress (CUMS) is a widely used depression model proposed by Willner in 1987 [11]. In the early experimental results of our group, we observed the decrease of E2 secretion and the increase of FSH in CUMS model rats, which was consistent with the clinical characteristics of POI. However, whether the expression of NGF and its receptors in ovarian tissue of CUMS animal models changes and plays a role in mediating the development of POI needs further observation and evaluation. Therefore, the present study investigated whether chronic stress is mediated by the NGF/TrkApathway and affects the ovarian follicular development leading to the onset of POI.

## 2. Materials and Methods

### 2.1. Animals

A total of 40 specific pathogen-free (SPF) female Sprague–Dawley rats (age, 12 weeks; no birth; average weight, 216 ± 12 g) were provided by the Experimental Animal Center of Zhejiang Academy of Medical Sciences (animal certificate number: SCXK (Zhejiang) 2014-0001). The housing methods of experimental animals are detailed in the previous study [6]. The experimental scheme of this study was approved by the Jinhua Polytechnic Medical Ethics Committee (Jinhua, PRC).

### 2.2. CUMS Rat Model

Animals were randomly divided into the control (*n* = 10) and CUMS groups (*n* = 30). Rats in the CUMS group were individually housed and repeatedly exposed to a set of the chronic unpredictable mild stressors. One stressor was randomly administered daily for 35 days. The method is described in detail in the previous research [6, 12].

### 2.3. Serum Detection

Blood samples (1 mL) were withdrawn from the posterior venous plexus of rats, after rats were anesthetized with pentobarbital (5 mg/100 g, intraperitoneal injection), on the days of 0, 14, and 35 of CUMS modeling, respectively. The estrous cycles of the rats were monitored continuously by vaginal smear. For rats with estrous cycle, serum hormones were detected in the proestrus. For rats without estrous cycle, serum hormones were detected at any time. After precipitation, the serum was centrifuged at 600 × g for 10 min, and the supernatant was collected. Then, according to the manufacturer's protocol, the serum levels of gonadotropin-releasing hormone (GnRH), follicle-stimulating hormone (FSH), estradiol (E2), and anti-Mullerian hormone (AMH) were detected using ELISA kits (Cusabio Technology, LLC, Wuhan, PRC) according to our previous description [6]. Curve Expert 1.3 software (Hyams Development, Huntsville, AL, USA) was used to draw standard curves, construct regression equations, and calculate the concentration of specific proteins in each sample.

### 2.4. Western Blotting

Rats were anesthetized (5 mg/100 g, intraperitoneal injection) and euthanized by cervical dislocation on day 35, and bilateral ovaries were collected for subsequent experiments. Ovarian tissues were treated with RIPA lysis buffer (Wuhan Boster Biological Technology, Ltd., Wuhan, PRC). The protein concentration of each sample was determined with a BCA protein quantification kit (Wuhan Boster Biological Technology, Ltd.). Membranes were blocked with 5% dried skimmed milk (Wuhan Boster Biological Technology, Ltd.). The antibodies used in the experiment mainly include rabbit polyclonal anti-NGF (1 : 1000), rabbit polyclonal anti-FSHR (1 : 500), rabbit polyclonal anti-TrkA (1 : 500), and rabbit polyclonal anti-p75 (1 : 500; all Abcam, Cambridge, UK), goat anti-rabbit IgG secondary antibody (1 : 5000, Thermo Fisher Scientific, Inc.). The protein contents in the sample were calibrated and quantified with GAPDH (1 : 1000, Abcam) as internal reference. Immunodetection was performed using SuperSignal™ West Dura Extended Duration substrate (Thermo Fisher Scientific, Inc., Waltham, MA, USA). The X-ray films were developed, images were captured, and Quantity One software (Bio-Rad, Hercules, CA, USA) was used to analyze the gray values. The specific method of this experiment is shown in our previous research [13].

### 2.5. Reverse Transcription-Quantitative Polymerase Chain Reaction (RT-qPCR)

After, rats were anesthetized and euthanized on the days 35 of CUMS modeling. The ovarian tissues of control and CUMS groups were collected in liquid nitrogen. The total RNA was extracted by TRIzol reagent (Invitrogen; Thermo Fisher Scientific, Inc). The concentration, purity, and quality of RNA were detected by spectrophotometry nanodrop 2000 (Thermo Fisher Scientific, Inc). Subsequently, total RNA (1 *μ*g) was reverse-transcribed into cDNA by the Bestar qPCR RT kit (DBI Bioscience, Ludwigshafen, RL, FRG). Quantitative PCR primers were designed by Primer Premier 6.0 and synthesized by GENEWIZ Biotech Co., Ltd. (Suzhou, PRC). The primer sequences are listed in Table 1. Power SYBR Green PCR Master Mix (Applied Biosystems, Inc.) was used for qPCR reactions performed with 7500 Real-Time PCR System (applied biosystems, Inc., USA). The amplification system and reaction conditions are shown in Table 2. Each sample was tested three times. The relative expression level of each gene is represented by *Δ*Cq, which is the difference between the internal control gene value and the CQ value of each sample. The 2-*ΔΔ*cq values were subsequently statistically analyzed.

### 2.6. Hematoxylin-Eosin Staining and Immunofluorescence

After, rats were anesthetized and euthanized on the days 35 of CUMS modeling. Ovarian tissues were fixed in 4% formaldehyde, dehydrated by an ethanol gradient, embedded in paraffin, sliced into serial 5 mm-thick sections, and then stained with hematoxylin for 5 min and eosin for 2 min. The numbers of each type of follicle were counted in unilateral ovaries in SD rats. Serial sections of each ovary were selected. Only those small follicles with nuclei visible in the oocyte are counted. The follicles were counted at 100 times magnification under the light microscope. The diameter of oocytes was measured to determine the classification of follicles. The follicle count method has been supplemented in the manuscript. According to the previous description [14], the follicles were detected and classified as small follicles, medium follicles, large follicles.

EDTA antigen retrieval buffer (pH 9.0; Beyotime Institute of Biotechnology, Shanghai, PRC) was used, and the sections were subsequently washed three times in PBS. A blocking solution was added for incubation at 37°C for 1 h. The following primary antibodies were subsequently used: rabbit polyclonal anti-NGF (1 : 100), mouse monoclonal anti-FSHR (1 : 100; Abcam), rabbit polyclonal anti-TrkA (1 : 100), and mouse monoclonal anti-p75 NGF (1 : 100; Abcam). The sections were incubated in a wet box at 4°C overnight, washed with PBS three times and incubated with the following secondary antibodies as follows; goat anti-mouse IgG (FITC) (1 : 50; Abcam) and goat anti-rabbit IgG (TRITC) (1 : 50; Abcam), in the dark at 37°C for 50 min. Subsequently, the samples were washed with PBS three times, dried, and incubated with the DAPI dye solution (100 ng/ml; Sigma-Aldrich, Merck KGaA, Darmstadt, FRG), at room temperature (20–25°C) in the dark for 10 min and washed with PBS three times. The slides were sealed and observed under a fluorescence microscope.

### 2.7. Ovarian Tissue Culture of CUMS Rats *in vitro*

As previously described, thirty rats of the CUMS model were anesthetized (5 mg/100 g, intraperitoneal injection) and euthanized by cervical dislocation. Bilateral ovarian tissues were taken under sterile conditions and cut into 2 mm × 2 mm × 2 mm under a stereomicroscope. The ovarian tissues were cultured in a 24-well culture plate containing 1 mL DMEM F-12 medium (Thermo Fisher Scientific, Inc.) with glucose (4.5 g/L; Thermo Fisher Scientific, Inc.), and penicillin-streptomycin (100 U/mL; Thermo Fisher Scientific, Inc.) in each well, and incubated at 39°C under 60% O_2_, 35% N_2_, and 5% CO_2_ [9, 15]. The tissues were randomly divided into four groups of six duplicate wells each. The control group was cultured in basal medium for 48 h, and the medium was changed after 24 h. The NGF group was cultured in medium with human recombinant *β*-NGF (200 ng/mL, Sigma-Aldrich, Merck KGaA) for 24 h, then replaced with basal medium for 24 h. The FSH group was cultured in basal medium for 24 h, then replaced with medium containing human FSH (500 ng/mL; Sigma-Aldrich, Merck KGaA) for 24 h. The NGF/FSH group was cultured in medium with human recombinant *β*-NGF (200 ng/ml) for 24 h, then replaced with medium containing human FSH (500 ng/ml) for 24 h. The tissues were collected for Western blotting and RT-qPCR tests, and E2 and AMH expressions of tissue culture supernatant were detected by ELISA, as previously described.

### 2.8. Statistical Analysis

All data are presented as the mean ± standard deviation and analyzed using SPSS statistical software (version 20.0; IBM Corp., Armonk, NY, USA). A *t*-test was used for comparisons between the two groups. Enumeration data were analyzed by the nonparametric Kruskal-Wallis test, using a nonparametric post hoc test (LSD test). The analysis of variance of factorial design was used in the statistical analysis of tissue culture experiment. *P* < 0.05 was considered to indicate a statistically significant difference.

## 3. Results

### 3.1. CUMS Reduces the Levels of Serum AMH, E2, and GnRH, and Increases the Level of FSH in Rats

**Figure 1:**
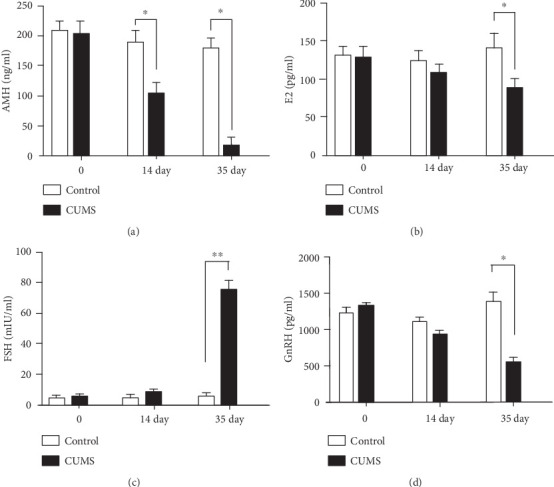
Effects of CUMS on hormone levels in rats. (a) The serum AMH level was affected by CUMS in rats at different points in time. (b) The serum E2 level was affected by CUMS in rats at different points in time. (c) The serum FSH level was affected by CUMS in rats at different points in time. (d) The serum GnRH level was affected by CUMS in rats at different points in time. Values are 10 in the control group and 30 in the CUMS group, respectively. ∗*P* < 0.05, and ∗∗*P* < 0.01 compared with the control group.

Before modeling, there was no significant difference between the two groups in the E2, AMH, GnRH, and FSH levels. The serum level of AMH was significantly lower in the CUMS group than in the control group on day 14 (*P* < 0.05). The levels of E2, AMH, and GnRH on day 35 were significantly lower (*P* < 0.05), while the levels of FSH were significantly higher in the CUMS group than in the control group (*P* < 0.01; Figure 1).

### 3.2. CUMS Reduces the Expression of NGF, Trk A, and FSHR in the Ovarian Tissues of CUMS Rats

**Figure 2:**
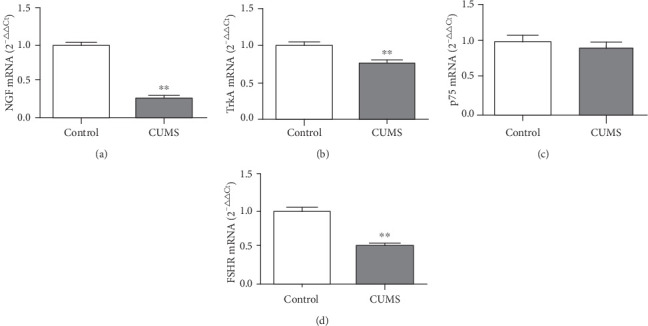
Effects of CUMS on the relative expression of NGF, TrkA, p75, and FSHR mRNA in the ovarian tissues of CUMS rats. (a) The NGF mRNA expression was affected by CUMS in the ovarian tissue of rats. (b) The TrkA mRNA expression was affected by CUMS in the ovarian tissue of rats. (c) The p75 mRNA expression was affected by CUMS in the ovarian tissue of rats. (d) The FSHR mRNA expression was affected by CUMS in the ovarian tissue of rats. Values are 10 in the control group and 30 in the CUMS group, respectively. ∗*P* < 0.05, ∗∗*P* < 0.01, compared with the control group.

**Figure 3:**
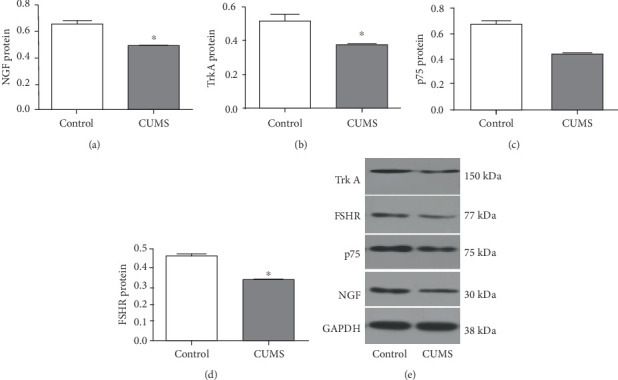
Effects of CUMS on the expression of NGF, TrkA, p75, and FSHR proteins in the ovarian tissues of rats. (a) The NGF protein expression was affected by CUMS in the ovarian tissue of rats. (b) The TrkA protein expression was affected by CUMS in the ovarian tissue of rats. (c) The p75 protein expression was affected by CUMS in the ovarian tissue of rats. (d) The FSHR protein expression was affected by CUMS in the ovarian tissue of rats. (e) Protein bands for the control and CUMS groups are shown. The molecular weight of TrkA is 150 kDa, FSHR is 77 kDa, p75 is 75 kDa, NGF is 30 kDa, and GAPDH is 38 kDa. Values are 10 in the control group and 30 in the CUMS group, respectively. ∗*P* < 0.05, ∗∗*P* < 0.01, compared with the control group.

The mRNA relative expression levels of NGF (0.26 ± 0.02), TrkA (0.75 ± 0.05), and FSHR (0.51 ± 0.03) were significantly lower in the ovarian tissue of CUMS rats than in the control group (*P* < 0.05). There was no significant difference in the expression level of p75 mRNA in the ovary of CUMS rats (0.92 ± 0.06) compared with the control group (*P* > 0.05; Figure 2). The protein expression levels of NGF (0.51 ± 0.01), TrkA (0.38 ± 0.01), and FSHR (0.40 ± 0.02) were significantly lower in the ovarian tissue of CUMS rats than in the control group, NGF (0.65 ± 0.01), TrkA (0.53 ± 0.00), and FSHR (0.47 ± 0.00) (*P* < 0.05). The protein expression level of p75 (0.50 ± 0.01) was slightly lower in the ovarian tissue of CUMS rats p75 (0.52 ± 0.01) (*P* > 0.05; Figure 3).

### 3.3. Localization of NGF, TrkA, p75, and FSHR Proteins in the Ovarian Tissue Determined Using Immunofluorescence

**Figure 4:**
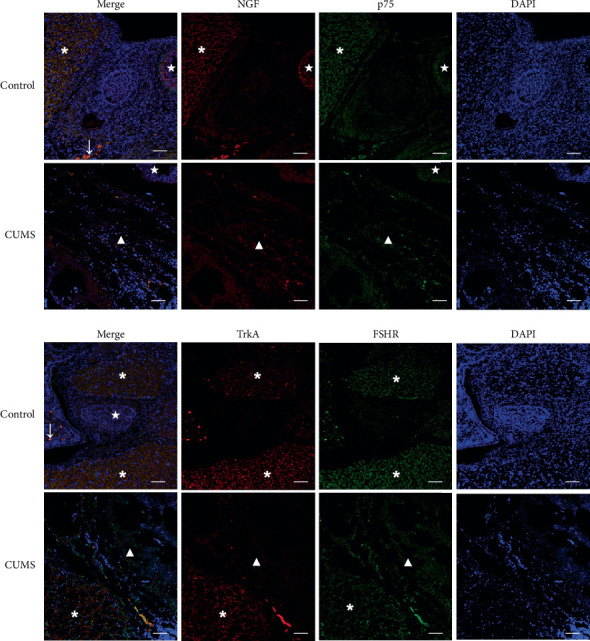
Immunofluorescence localization of NGF, p75, TrkA, and FSHR proteins in the ovarian tissues of CUMS rats Tissues were stained for NGF using TRITC-labeled secondary antibody (red), and for p75 using FITC-labeled secondary antibody (green). Nuclei were stained using DAPI (blue). Tissues were stained for TrkA using TRITC-labeled secondary antibody (red) and for FSHR using FITC-labeled secondary antibody (green). Nuclei were stained using DAPI (blue). In the figure, the ∗ symbol indicates corpus luteum, ★ indicates antral follicles, ↓and small follicles, and ▲ indicates ovarian stroma, scale bar = 50 *μ*m. Values are 10 in the control group and 30 in the CUMS group, respectively.

NGF, TrkA, p75, and FSHR proteins were expressed in the ovarian tissues of CUMS rats to varying degrees, detected by immunofluorescence. NGF protein was mainly expressed in luteal cells, granulosa cells, and theca cells of antral follicles and oocytes of small follicles. FSHR protein was mainly expressed in theca cells and oocytes of antral follicles. p75 protein was expressed in oocytes of small follicles, theca cells of antral follicles, and luteal cells. TrkA protein was expressed in granulosa cells of antral follicles and oocytes of small follicles (Figure 4).

### 3.4. The Number of Small Follicles Decreases in CUMS Rats

**Figure 5:**
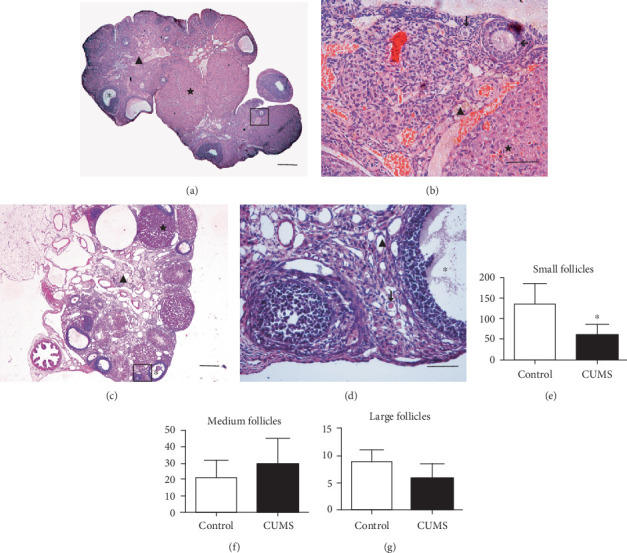
Morphological alterations in the ovarian tissues of CUMS rats. (a) Control group, there are many normal corpus luteum (★) and large follicles (∗) in ovarian tissue. (b) A close-up section of the image (a) there are many normal small follicles (↓) and medium follicles (←) in ovarian tissue (▲). (c) In the CUMS group, the morphological structure of the ovary shows obvious disorders, including ovarian cortex thickening, ovarian interstitial fibrosis, follicular reduction, and corpus luteum (★) atrophy. (d) A close-up section of image (c), the number of small follicles (↓) decreased, the difference was statistically significant, there were large amounts of atretic large follicles (∗). Scale bar = 100 *μ*m (A, C), scale bar = 50 *μ*m (b, d). (e) Small follicles in control group and CUMS group. (f) Medium follicles in control group and CUMS group. (g) Large follicles in control group and CUMS group. Values are 10 in the control group and 30 in the CUMS group, respectively. ∗*P* < 0.05 vs. control group. CUMS: chronic unpredictable mild stress.

In the CUMS group, the ovarian interstitial structure was disordered, and severe fibrosis, cortical thickening, and corpus luteum fibrosis were identified (Figure 5). The number of small follicles obtained by serial sections in the CUMS group was 58.90 ± 26.25, which was significantly lower than in the control group (137.13 ± 48.55; *P* < 0.01). The number of middle follicles in the control group was 20.88 ± 11.44, and that in the CUMS group was 29.30 ± 15.79; the number of large follicles in the control group was 9.00 ± 2.27, and that in the CUMS group was 6.00 ± 2.75. There was no significant difference between the two groups (*P* > 0.05).

### 3.5. Exogenous NGF Upregulates the Expression of TrkA, p75, and FSHR of CUMS Rat Ovarian Tissue *in vitro*

**Figure 6:**
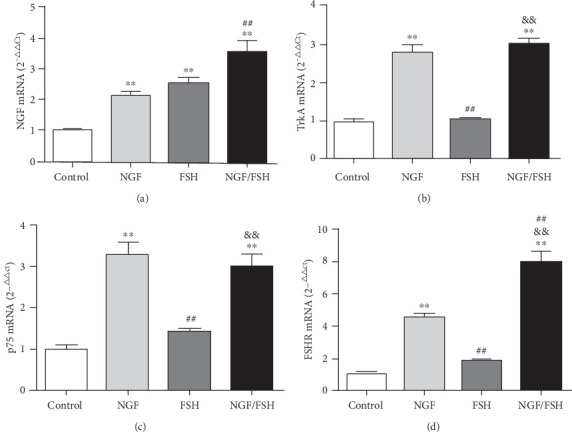
The relative expression of TrkA, p75, and FSHR mRNA of CUMS rats ovarian tissue was upregulated by Exogenous NGF *in vitro*. (a) Variations of NGF mRNA expression in the ovarian tissue of CUMS rats after intervention with exogenous NGF *in vitro*. (b) Variations of TrkA mRNA expression in the ovarian tissue of CUMS rats after intervention with exogenous NGF *in vitro*. (c) Variations of p75 mRNA expression in ovarian tissue of CUMS rats after intervention with exogenous NGF *in vitro*. (d) Variations of FSHR mRNA expression in ovarian tissue of CUMS rats after intervention with exogenous NGF *in vitro*. Values are mean ± SD for six samples. ∗*P* < 0.05, ∗∗*P* < 0.01, compared with the control group; ^#^*P* < 0.05, ^##^*P* < 0.01 compared with the NGF group; ^&^*P* < 0.05, ^&&^*P* < 0.01, compared with the FSH group.

**Figure 7:**
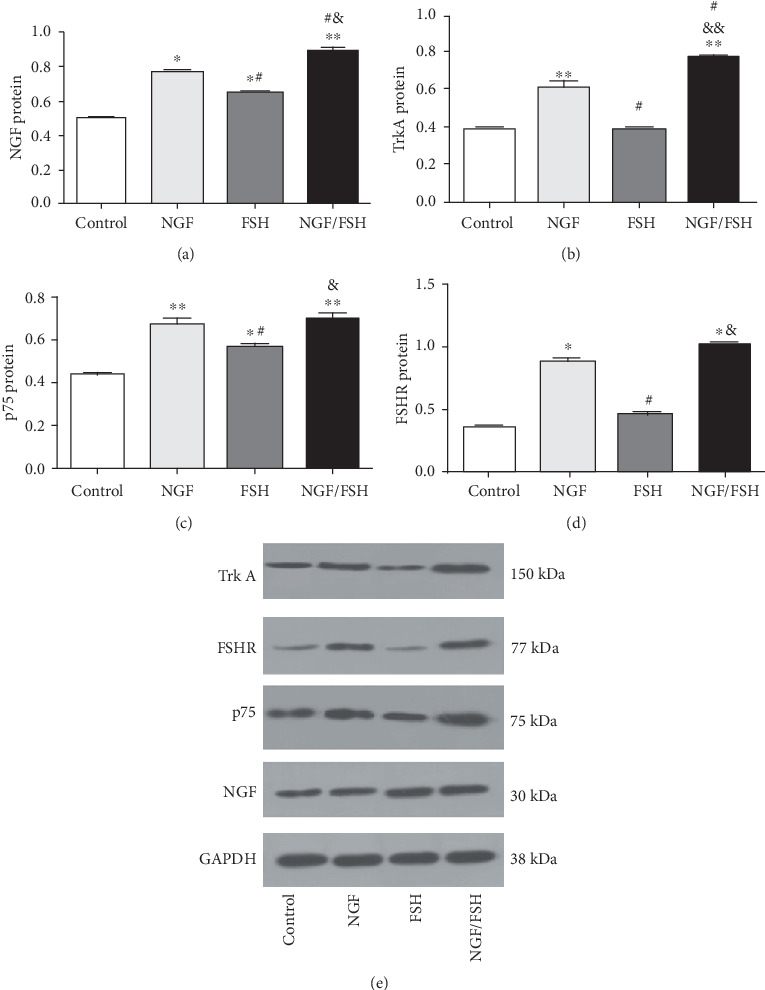
The expression of TrkA, p75, and FSHR proteins was upregulated by exogenous NGF in ovarian tissue of CUMS rats *in vitro*. (a) Variations of NGF protein expression in the ovarian tissue of CUMS rats after intervention with exogenous NGF *in vitro*. (b) Variations of TrkA protein expression in the ovarian tissue of CUMS rats after intervention with exogenous NGF *in vitro*. (c) Variations of p75 protein expression in the ovarian tissue of CUMS rats after intervention with exogenous NGF *in vitro*. (d) Variations of FSHR protein expression in the ovarian tissue of CUMS rats after intervention with exogenous NGF *in vitro*. (e) Protein bands for the control, NGF, FSH, and NGF/FSH groups are shown. The molecular weight of TrkA is 150 kDa, FSHR is 77 kDa, p75 is 75 kDa, NGF is 30 kDa, and GAPDH is 38 kDa. Values are mean ± SD for six samples. ∗*P* < 0.05, ∗∗*P* < 0.01, compared with the control group; ^#^*P* < 0.05, ^##^*P* < 0.01 compared with the NGF group; ^&^*P* < 0.05, ^&&^*P* < 0.01, compared with the FSH group.

The expression levels of NGF, TrkA, p75, and FSHR mRNA in the NGF and NGF/FSH groups were significantly higher than those in the control group (*P* < 0.01), and only NGF mRNA in the FSH group was significantly higher than in the control group (*P* < 0.01). The expression of NGF and FSHR mRNA in the NGF/FSH group was significantly higher than in the NGF group (*P* < 0.01; Figure 6). The expression levels of NGF (*P* < 0.05, *P* < 0.01), TrkA (*P* < 0.01), p75 (*P* < 0.01), and FSHR (*P* < 0.05, *P* < 0.01) protein in the NGF and NGF/FSH groups were significantly higher than those in the control group. Only the expression of NGF and p75 protein in the FSH group was significantly higher than that in the control group (*P* < 0.05). The expression of NGF and TrkA protein in the NGF/FSH group was significantly higher than in the NGF group (*P* < 0.05; Figure 7).

### 3.6. Exogenous NGF Induces Changes in the Secretion of AMH and E2 in CUMS Rat Ovarian Tissue

**Figure 8:**
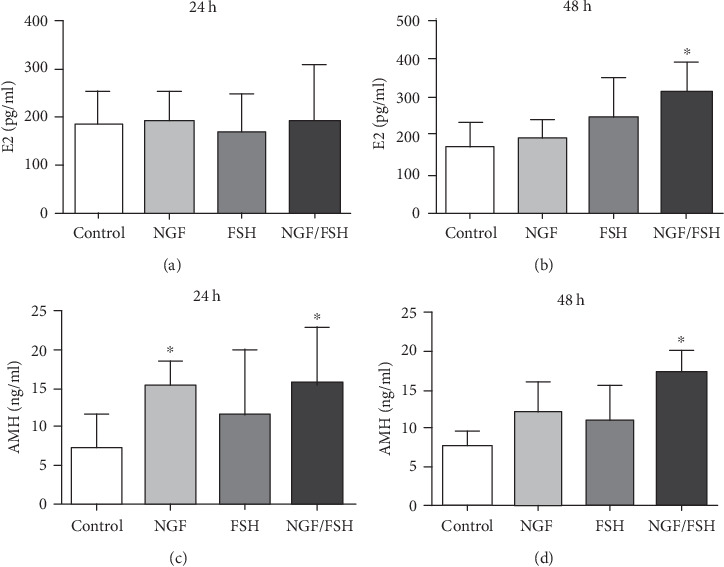
Effects of exogenous NGF on the secretion of E2 and AMH in the ovarian tissue of CUMS rats at different intervals *in vitro*. (a) Changes of E2 secretion were induced by Exogenous NGF in the ovarian tissue of CUMS rats at 24 h. (b) Changes of E2 secretion were induced by Exogenous NGF in the ovarian tissue of CUMS rats at 48 h. (c) Changes of AMH secretion were induced by Exogenous NGF in the ovarian tissue of CUMS rats at 24 h. (d) Changes of AMH secretion were induced by Exogenous NGF in the ovarian tissue of CUMS rats at 48 h. Values are mean ± SD for six samples. ∗*P* < 0.05, compared with the control group.

There was no significant difference in E2 secretion when CUMS rat ovarian tissues were cultured for 24 h. However, the secretion of E2 in the NGF/FSH group was significantly increased at 48 h (*P* < 0.05). The AMH secretion of the NGF and NGF/FSH groups was higher than in the control group at 24 h. At 48 h, the AMH secretion of the NGF/FSH group was higher than in the control group, and the difference was statistically significant (*P* < 0.05; Figure 8).

## 4. Discussion

Psychological stress can inhibit and impair the female reproductive and endocrine functions through the hypothalamic-pituitary-gonadal axis. These changes may lead to a decline in ovarian reserve and POI. The mechanisms underlying ovarian dysfunction and POI caused by chronic stress remain unclear and may be associated with NGF [9]. Serum levels of E2, FSH, GnRH, and AMH can accurately reflect alterations in ovarian function and may be used for the evaluation of animal models of POI. Animal models of POI exhibit decreased and increased levels of E2 and FSH, respectively.

Furthermore, AMH is an indicator for the evaluation of the ovarian reserve [16–18]. Lu et al. [19] found the levels of E2, and other hormones were reduced in CUMS rats and stress-induced a disordered estrus cycle. Chronic cold stress can lead to ovarian dysfunction or decline in rats, prolonged sexual cycles or no physiological cycle, and E2 and progesterone secretion disorders [10]. In our previous research, we found that the body weight of CUMS rats decreased, sugar consumption and preference reduced, and vertical and horizontal movement in the open field decreased [6]. The results of the present study indicate that serum E2 levels decrease in the CUMS group, while FSH and GnRH levels increase, which is consistent with the characteristics of POI described in previous studies.

Furthermore, the AMH, indicative of ovarian reserve function, was significantly reduced in the rat models of CUMS used in the present study. The comprehensive analysis of serum E2, FSH, and AMH indicated that the rat models of CUMS exhibited signs of ovarian dysfunction. The alterations in these hormone levels also indicated that CUMS could affect the hypothalamic-pituitary-gonadal axis, eventually leading to a decline in the ovarian function.

Immunofluorescence experiments indicate that the normal ovarian NGF and its receptor were expressed in follicular oocytes in all stages of development, and in the membrane, granulosa, luteal, and stromal cells. Ovarian NGF production was enhanced in granulosa cells and decreased in stromal cells, which was consistent with the findings of a study by Shi et al. [20]. By contrast, NGF expression in the CUMS group decreased compared with that in the control group. The TrkA receptor predominantly activates phosphatidylinositol-3-kinase (PI3K) and mitogenic activated protein kinase (MAPK) to promote cell survival and proliferation [7]. In both oocytes and granulosa cells, a complete PI3K/Akt signaling system coordinates and regulates follicular growth, maturation, and periodic ovulation [21]. The PI3K/Akt signaling pathway regulates oocyte growth, and the survival and development of primordial follicles promote the proliferation and differentiation of granulosa cells and inhibits apoptosis, which is critical for the normal development and physiological functions of the ovaries [22, 23]. In the current study, the mRNA and protein expression levels of NGF and TrkA were lower in the CUMS group than in the control group, suggesting that chronic stress can downregulate the expression of NGF and its receptors in the rat ovarian tissue. In addition, TrkA and p75 proteins were expressed in the ovarian stroma and small follicle oocytes by immunofluorescence. The results indicate that NGF and its receptor play an important role in early follicular development. Therefore, we speculate that NGF/TrkA may be involved in the initiation of primordial follicle growth.

TrkA and p75 exhibit both synergistic and antagonistic effects, and the underlying regulatory mechanisms remain unclear. The binding of NGF to TrkA can increase the effective concentration of NGF on the cell surface in the presence of p75 and increase its affinity by 25-fold [24]. In the absence of TrkA, the binding of NGF to p75 could induce apoptosis [25]. In addition, the number of primary and secondary follicles increased in p75 knockout mice [26]. One research group has hypothesized that p75 may act as a modulator in pre-thecal mesenchymal cells and regulate follicle activation [7]. The results showed that CUMS significantly decreased the expression of NGF, TrkA, and FSHR in the ovarian tissue of rats, but p75 did not. Because of the significant decrease of the TrkA expression level, NGF cannot bind to TrkA but can bind to P75, which promotes the apoptosis of a large number of small follicles. This also explains why the number of small follicles in ovarian tissue of CUMS rats decreases, and the release of AMH and E2 from ovarian tissue decreases. In conclusion, CUMS may affect ovarian function and contribute to premature ovarian failure through NGF and its receptor-mediated signaling pathway.

In order to further verify whether the effect of CUMS on ovarian function is mediated by the NGF/TrkA signaling pathway, *in vitro* culture experiments were conducted to observe the expression of NGF and its receptors in the ovarian tissues of CUMS and the changes of ovarian function after the intervention of exogenous NGF. The results showed that the expression levels of NGF, TrkA, p75, and FSHR were upregulated after the intervention of beta-NGF, especially TrkA and FSHR. These changes may be related to the paracrine or autocrine effects of NGF. NGF can not only promote its expression but also induce the increase of its receptors TrkA and p75 [27]. NGF interacts with its receptors p75 and TrkA and affects their conformation. When NGF binds to the receptor, it can induce dimerization. It was found that the expression of TrkA and p75 in ovarian tissue increased simultaneously, which would promote the formation of TrkA homologous dimer and p75 isomer. TrkA homologous dimer and p75 isomer can promote cell survival, and p75 homologous dimer mediates cell apoptosis [28]. It can be inferred that NGF is the nutritional support of ovarian sympathetic innervation, and it directly affects follicular survival through NGF/TrkA signal transduction.

In the present study, NGF and FSH were added to the culture medium, the secretion of AMH and E2 was found to be increased at 48 h, which indicates that NGF improves the expression of FSHR and enhances FSH reactivity, thereby promoting the secretion of E2 and the development of follicles (especially antral follicles). In addition to the direct effects mentioned above, NGF can also play an indirect role in promoting follicle survival by producing follicle hormone receptor (FSHR). After NGF intervention, the expression of FSHR in granulosa cells increased [15], and the response to FSH was enhanced [29, 30]. The upregulation of FSHR expression in the ovarian tissue of CUMS after NGF intervention was also observed in this study. The expression of NGF and TrkA in the ovarian tissue of CUMS was further increased by adding FSH after NGF intervention, and the content of E2 in ovarian tissue increased significantly 48 hours after culture. This suggests that the ovarian tissue after NGF treatment may enhance the expression of FSHR, thereby increasing the responsiveness to FSH, thus promoting follicular growth, development, and E2 secretion.

The data of this experiment show that exogenous NGF can improve the ovarian dysfunction caused by CUMS. However, previous studies have found that high concentrations of NGF may be harmful to the female reproductive system [31]. Due to the overproduction of stathmin within the ovarian tissue, the apoptotic rate of granulosa cells increases in the ovaries of transgenic animals with the excessive expression of NGF [32]. NGF may also exert a stimulatory effect in ovarian cancer and polycystic ovarian syndrome [33]. Current research cannot explain the above contradictions. Although current experimental data confirm that the development of POI induced by CUMS is related to NGF and its receptor-mediated pathway, many details remain unclear. For example, the expression of TrkA and P75 receptors in different stages of follicular development and different types of ovarian tissue cells (oocyte, granulosa cell, or follicular membrane cell), the expression of downstream genes caused by the NGF/TrkA signaling pathway, and the dose-dependent relationship between POI and NGF expression. These problems require further study and exploration.

In conclusion, CUMS downregulates NGF and TrkA and promotes the occurrence of POI in rats. Exogenous NGF and FSH can upregulate the NGF receptor, E2, and AMH *in vitro*, and improve the rat ovarian function. Future studies may associate these results with female population.

## Figures and Tables

**Table 1:** Specific primer sequence designed by Primer Premier 6.0.

Gene	GenBank accession	Primer sequence (5′-3′)
Rat FSHR	NM_199237.1	GGATGGCCTTGCTCCTGGTC
		GGGAGGTCGGTCGGAATCTC
Rat TrkA	NM_021589.1	TGCCCTCCTCCTAGTGCTCA
		CGGAGCCTTTGCCCTCAGTA
Rat NGF	NM_001277055.1	TGCACCACGACTCACACCTT
		TGCAGGCAAGTCAGCCTCTT
Rat p75	NM_130426.4	CCTACCTCAGCCTGGAGCAC
		CTGACGCCATTGGCTGTTCC
Rat GAPDH	M17701.1	TGATTCTACCCACGGCAAGT
		AGCATCACCCCATTTGATGT

**Table 2:** Real-time PCR amplification system and reaction conditions.

Reagent component	20 *μ*L system	Reaction conditions
SDW	8.0 *μ*L	95°C, 1 min; (95°C, 15 sec, 60°C, 40 sec) 40 cycles; 55 to 95°C melting curve
Power SYBR green master mix	10.0 *μ*L	
Forward primer(10 *μ*M)	0.5 *μ*L	
Reverse primer(10 *μ*M)	0.5 *μ*L	
cDNA	1.0 *μ*L	

## Data Availability

The data used to support the findings of this study are available from the corresponding author upon request.

## Abbreviations

POI: Premature ovarian insufficiency
CUMS: Chronic unpredictable mild stress
AMH: Anti-Mullerian hormone
GnRH: Gonadotropin-releasing hormone
FSH: Follicle-stimulating hormone
FSHR: Follicle-stimulating hormone receptor
E2: Estradiol
NGF: Nerve growth factor
TrkA: Tropomyosin-related kinase A
RT-qPCR: Reverse transcription-quantitative polymerase chain reaction.

## Supplementary Materials

Supplementary Materials Figure S1: effects of CUMS on the expression of NGF, Trk A, p75, FSHR proteins in the ovarian tissues of rats. Figure S2: the expression of TrkA, p75, and FSHR proteins was upregulated by exogenous NGF in ovarian tissue of CUMS rats *in vitro*.
