# Supplementary material for: CUMS Promotes the Development of Premature Ovarian Insufficiency Mediated by Nerve Growth Factor and Its Receptor in Rats
Source: Biomed Res Int. 2020 Jun 30;2020:1946853. doi: 10.1155/2020/1946853 (PMC7345596; doi:10.1155/2020/1946853)
Supplement: Supplementary Materials — Figure S1: effects of CUMS on the expression of NGF, Trk A, p75, FSHR proteins in the ovarian tissues of rats. Figure S2: the expression of TrkA, p75, and FSHR proteins was upregulated by exogenous NGF in ovarian tissue of CUMS rats in vitro. [file 1946853.f1.pdf]

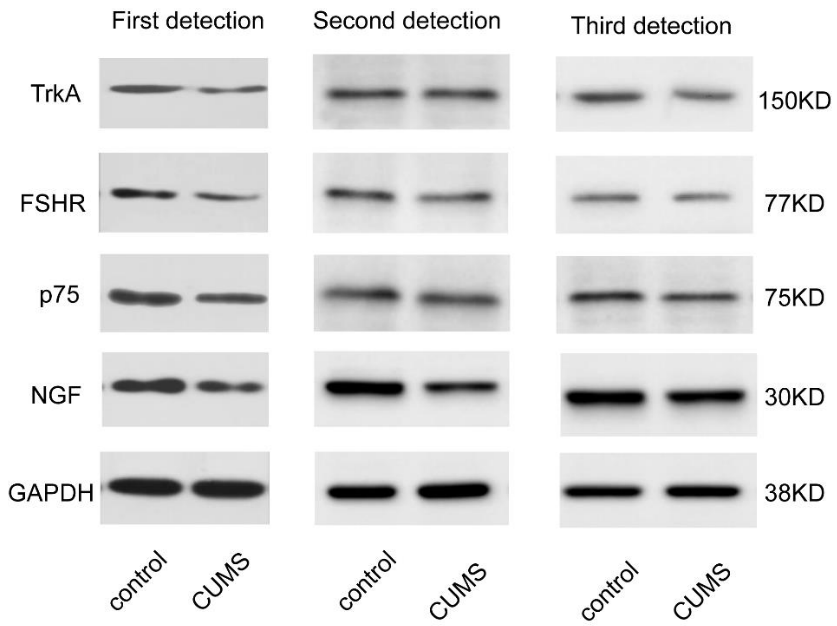

Figure S1 Effects of CUMS on the expression of NGF, Trk A, p75, FSHR proteins in the ovarian tissues of rats

The effect of CUMS on the expression of various proteins in rat ovary is shown in the figure. Weston blot was used to detect the proteins in ovarian tissue of control group and CUMS group respectively, and the samples of each group were detected three times. The gray band of protein obtained by three tests is shown in the figure. The molecular weights of the four proteins were Trk A 150 kD, FSHR 77 kD, p75 75 kD and NGF 30 kD, respectively, and GAPDH was 38 kD.

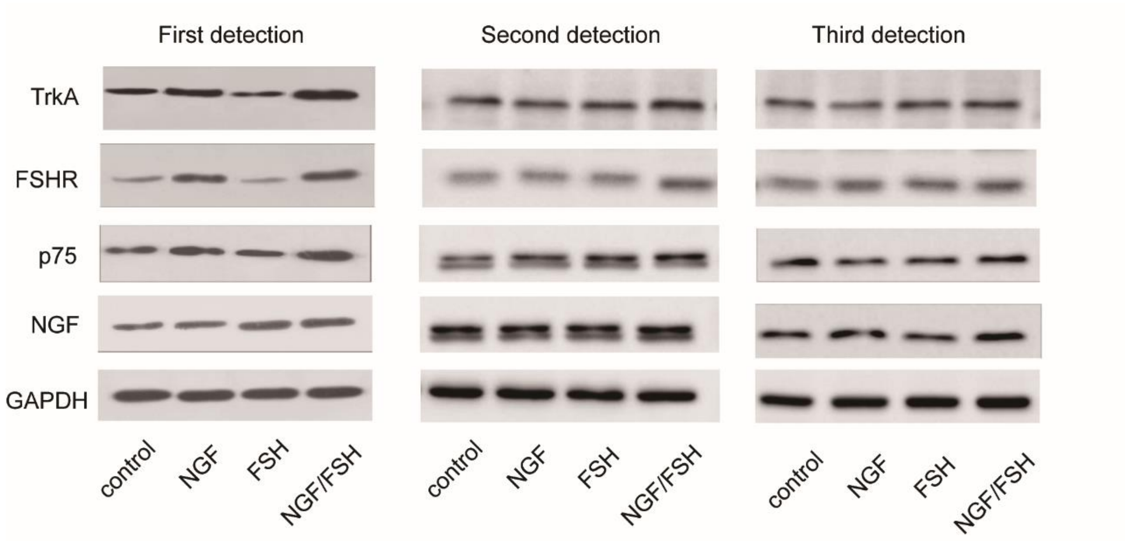

Figure S2 The expression of TrkA, p75, and FSHR proteins was upregulated by exogenous NGF in ovarian tissue of CUMS rats *in vitro*

The picture shows the protein expression of CUMS ovarian tissue in vitro induced by exogenous NGF. Weston blot was used to detect the proteins in ovarian tissue samples of four groups, including control group, NGF group, FSH group and NGF / FSH group. The samples of each group were repeatedly detected three times. The gray band of protein obtained by three tests is shown in the figure. The molecular weight of the four proteins were Trk A 150 kD, FSHR 77 kD, p75 75 kD, NGF 30 kD, and GAPDH 38 kD.
